# Whole genome and exome sequencing of monozygotic twins discordant for Crohn’s disease

**DOI:** 10.1186/1471-2164-15-564

**Published:** 2014-07-05

**Authors:** Britt-Sabina Petersen, Martina E Spehlmann, Andreas Raedler, Björn Stade, Ingo Thomsen, Raquel Rabionet, Philip Rosenstiel, Stefan Schreiber, Andre Franke

**Affiliations:** Institute of Clinical Molecular Biology, Christian-Albrechts-University of Kiel, Schittenhelmstrasse 12, 24105 Kiel, Germany; Department of Internal Medicine, University Hospital Schleswig-Holstein, Kiel, Germany; Department of Internal Medicine II, Gastroenterology, Asklepios Westklinikum Hamburg, Hamburg, Germany; Genomics and Disease Group, Center for Genomic Regulation, Barcelona, Spain

**Keywords:** Crohn’s disease, Discordant monozygotic twins, Somatic mosaicism, Whole genome sequencing, Exome sequencing

## Abstract

**Background:**

Crohn’s disease (CD) is an inflammatory bowel disease caused by genetic and environmental factors. More than 160 susceptibility loci have been identified for IBD, yet a large part of the genetic variance remains unexplained. Recent studies have demonstrated genetic differences between monozygotic twins, who were long thought to be genetically completely identical.

**Results:**

We aimed to test if somatic mutations play a role in CD etiology by sequencing the genomes and exomes of directly affected tissue from the bowel and blood samples of one and the blood-derived exomes of two further monozygotic discordant twin pairs. Our goal was the identification of mutations present only in the affected twins, pointing to novel candidates for CD susceptibility loci. We present a thorough genetic characterization of the sequenced individuals but detected no consistent differences within the twin pairs. An estimate of the CD susceptibility based on known CD loci however hinted at a higher mutational load in all three twin pairs compared to 1,920 healthy individuals.

**Conclusion:**

Somatic mosaicism does not seem to play a role in the discordance of monozygotic CD twins. Our study constitutes the first to perform whole genome sequencing for CD twins and therefore provides a valuable reference dataset for future studies. We present an example framework for mosaicism detection and point to the challenges in these types of analyses.

**Electronic supplementary material:**

The online version of this article (doi:10.1186/1471-2164-15-564) contains supplementary material, which is available to authorized users.

## Background

Crohn’s disease (CD, OMIM #266600) is a complex, chronic inflammatory bowel disease (IBD) affecting between 0.1–16/100,000 persons worldwide, with a higher incidence in the Western world. As a multifactorial disease, a variety of genetic and environmental factors play a role in its etiology. Genome-wide association studies (GWAS) and meta-analyses have so far identified a total of 163 susceptibility loci for IBD [1], with 140 for CD. These GWAS loci have highlighted important pathways underlying IBD, such as immunity and autophagy. Yet, the identified variants so far explain less than 30% of the cumulative genetic variance for CD and it is thought that a part of the so-called missing heritability may be found in rare variants with larger effect sizes. Next-generation sequencing enables the genome- and exome-wide identification of novel variants and is therefore the current method of choice for finding new rare susceptibility loci for complex diseases. In the past 5 years researchers even identified several monogenic forms of severe early-onset colitis. For example, single mutations in *IL10* (interleukin-10 [2]) and the genes encoding for its receptor (*IL10RA* and *IL10RB* [3]) as well as mutations in *XIAP* (X-linked inhibitor of apoptosis protein [4, 5]) have been shown to cause severe early-onset IBD. Monozygotic (MZ) twins have long served as a model for the influence of environmental factors versus genetic factors (“nature vs. nurture”), since they are believed to be genetically identical. For example, our previously published German epidemiologic twin study revealed that red meat consumption, high antibiotic intake and living abroad before time of diagnosis, especially in countries of the non-developed world, may play a role in disease etiology [6]. Moreover, our group identified several differentially methylated sites in the colonic epigenome of discordant colitis twins with functional consequences, i.e. impact gene expression [7]. Monozygotic discordant UC (Ulcerative colitis) twins were also shown to differ in the bacterial composition of their gut microbiota, with the affected twins showing less diversity than their healthy co-twins [8]. This suggests an important link between disease and the microbiome.

In the past years it has been repeatedly shown that the assumption of genetically identical monozygotic twins needs to be reconsidered. Genetic differences have been discovered between monozygotic twins [9], which can be regarded as an extreme form of somatic mosaicism, describing the presence of two populations of cells with different genotypes having developed from a single fertilized egg as a result of postzygotic alterations of the genome [10]. Several studies have shown discordant phenotypes between monozygotic twins resulting from genetic differences such as chromosomal mosaicism (e.g. trisomy 21 [11]) or dominant gene mutations (first shown for Van der Woude syndrome [12]) and differences in the copy number profiles of monozygotic concordant and discordant twin pairs were detected [13]. These findings challenge the assumption of disease discordance in monozygotic twins reflecting purely environmental effects. The genetic comparison of monozygotic twins discordant for a complex disease has been previously proposed for finding disease-relevant variants in a set of candidate genes [14]. During the past years sequencing costs have dropped dramatically, thus allowing for whole genome- and exome-wide comparisons of monozygotic twins, enabling the identification of these rare genetic events without the need for prior assumptions like focusing on certain candidate genes. The genetic comparison of monozygotic discordant twins therefore represents a promising possibility for finding novel candidates for disease susceptibility that may help explain some part of the missing heritability. Considering the estimated human mutation rate of ~2.2 to ~2.5×10^−8^ per position per diploid genome [15, 16], approximately 175 mutations per genome are expected per generation, indicating that genetic differences between monozygotic twins may not be so rare. However, whole genome sequencing and the comparison of monozygotic twins discordant for multiple sclerosis yielded no differences [17]. As a consequence, we aimed for a higher sequencing depth (per base coverage) in our study and included samples of the directly affected tissue in addition to blood samples in the sequencing to maximize our chances of detecting relevant somatic mutations.

**Figure 1:**
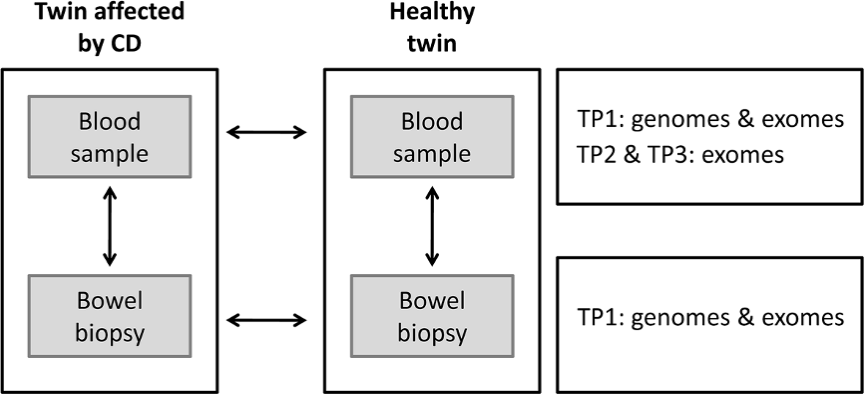
**Overview of study setup involving exome and genome sequencing for three monozygotic twin pairs (TP1-3) discordant for Crohn’s disease.**

We here report the sequences of four genomes and eight exomes of blood samples and bowel biopsies from three monozygotic twin pairs discordant for CD, the study setup is illustrated in Figure 1. From the German IBD twin cohort described in [18] we chose three twin pairs based on preferably low age of onset, high current age and the availability of blood samples and bowel biopsies (Table 1). By sequencing different sample types we were not only able to scan for differences between the twins, but additionally search for tissue-specific variants in the bowel tissue directly affected by the inflammation of CD. This is to our knowledge the first characterization of genomes and exomes of monozygotic twins discordant for Crohn’s disease.

**Table 1:** **Description of the three twin pairs and corresponding sequencing statistics**

	Twin pair 1								Twin pair 2		Twin pair 3	
Sex	Female								Female		Female	
Age of onset in affected twin	45								19		12	
Year of birth	1944								1962		1974	
Recruited	2007								2007		2006	
Last recontact	2008								2007		2012	
	**Genomes**				**Exomes**				**Exomes**			
	**Blood CD**	**Biopsy CD**	**Blood healthy**	**Biopsy healthy**	**Blood CD**	**Biopsy CD**	**Blood healthy**	**Biopsy healthy**	**Blood CD**	**Blood healthy**	**Blood CD**	**Blood healthy**
Gb coverage	126.70	124.02	143.82	110.28	3.44	3.16	2.47	3.50	3.33	3.29	2.86	2.21
Average coverage	41.92	41.03	47.58	36.49	55.36	50.83	39.81	56.34	72.29	71.58	62.22	47.88
% of genome/exome covered:												
≥ 1×	93.12	93.05	93.04	93.03	97.10	97.17	96.25	96.97	97.28	97.16	96.64	95.36
≥ 8×	90.46	88.95	88.77	90.49	91.48	90.72	89.11	91.82	90.13	89.87	88.91	85.25
≥ 20×	73.29	65.11	67.26	80.82	83.37	79.47	76.78	84.72	80.39	79.87	78.27	71.02
Total number of variants	3,064,772	3,053,010	3,055,001	3,081,680	44,890	44,357	44,928	44,920	29,180	28,985	28,407	27,465
Ti/Tv ratio	2.1	2.1	2.1	2.1	2.6	2.6	2.6	2.6	2.7	2.7	2.7	2.8
NS/S ratio	0.9	0.9	0.9	0.9	0.8	0.8	0.8	0.8	0.9	0.9	0.9	0.9
Total number of SNVs	2,894,614	2,878,835	2,877,807	2,911,350	44,377	43,846	44,426	44,406	28,663	28,542	27,947	27,185
Total number of InDels	170,158	174,175	177,194	170,330	514	512	503	515	517	443	460	280
Synonymous SNVs	9,655	9,630	9,658	9,625	10,168	10,046	10,163	10,205	8,977	8,920	8,678	8,457
Missense SNVs	8,443	8,414	8,425	8,413	8,600	8,488	8,588	8,618	7,668	7,639	7,562	7,369
Nonsense SNVs	64	66	63	65	57	56	57	57	56	58	58	54
Non-frameshift InDels	77	76	78	78	208	208	201	209	205	182	169	111
Frameshift InDels	53	48	54	48	144	142	141	143	296	246	273	162
Stopgain InDels	0	0	0	0	3	3	3	3	0	0	0	0
Splice-site variants	55	56	56	58	96	96	96	98	45	45	49	34
Variants in UTR	208,685	207,740	207,924	209,726	23,714	23,446	23,770	23,685	1,253	1,241	1,195	1,143
Intronic variants	956,711	953,878	954,959	962,132	1,659	1,635	1,667	1,659	10,154	10,127	9,836	9,573
Intergenic variants	1,881,029	1,873,102	1,873,784	1,891,535	242	238	243	244	526	527	587	562
Novel SNVs	54,823 (1.9%)	54,251 (1.9%)	54,372 (1.9%)	55,243 (1.9%)	988 (2.2%)	988 (2.3%)	960 (2.2%)	995 (2.2%)	2,009 (7.0%)	2,023 (7.1%)	1,802 (6.4%)	1,724 (6.3%)
Damaging SNV predictions												
HGMD	57	59	55	59	61	61	61	61	44	43	40	41
SIFT	697	689	697	695	685	686	683	689	674	670	645	630
PolyPhen2	734	731	728	733	765	764	755	767	653	651	648	639
Grantham score	1,442	1,437	1,445	1,439	1,496	1,499	1,477	1,501	1,356	1,355	1,372	1,327
PhyloP	7,753	7,728	7,730	7,757	1,854	1,855	1,837	1,859	1,968	1,964	1,957	1,892

Coverage and variant calls for exomes refer to the corresponding exome target. T_i_/T_v_ ratio: transition/transversion ratio of SNVs; NS/S ratio: nonsynonymous to synonymous ratio of SNVs; SNVs not in dbSNP132 for TP1 (hg19) and dbSNP130 for TP2 and TP3 (hg18) are defined as novel. SNVs predicted to be damaging: “DM” in HGMD (Human Gene Mutation Database Professional, version 2013.3), missense SNVs called as “deleterious” by SIFT, “probably damaging” by PolyPhen2, grantham score above 100 or positions with a phyloP value above 2.5.

All three twin pairs were female and of German ancestry and co-twins grew up together. In all affected twins, the disease showed ileocaecal localization and the diagnosis was confirmed by both endoscopy and histopathology. The first twin pair (TP1) was 63 years old at time of recruitment with an age-of-onset of 45 in the affected individual. The patient did not suffer from fistula or stenosis but presented with arthritis as an extraintestinal manifestation. So far, no surgery was necessary and flares occurred approximately once a year with few hospitalizations. The second twin pair (TP2) was 45 years old at time of recruitment. The affected twin had been suffering from Crohn’s disease since the age of 19 and presented with anal fistula to the skin and arthritis as an extraintestinal manifestation. She underwent emergency gut surgery due to ileal perforation in the ninth year of disease. The patient was hospitalized approximately once per year since disease onset and has in total spent more than 6 months in the hospital. Since surgery, she has no longer been suffering from regular flares. The third twin pair (TP3) was 32 years old at time of first participation in the study and showed the earliest age-of-onset of the three twin pairs with only twelve years. The patient presented with fistula (both anal and inguinal) and had been admitted to the hospital less than every two years for a total of one to three months since disease onset. The frequency of flares was one to three per year with few hospital stays necessary. In all twin pairs, the healthy twin showed no signs of intestinal inflammation.

## Results

For TP1, four genomes and four exomes of blood-derived DNA as well as DNA from bowel biopsies were sequenced. From TP2 and TP3, blood samples of both twins were exome sequenced, resulting in a total of four genomes and eight exomes from three twin pairs. All samples were sequenced to a minimum average coverage of 36× (Table 1). In total, more than 500Gb of sequences were uniquely aligned for the four genomes, resulting in coverage of more than 93% of the whole human genome reference. This value represents the upper limit of what can possibly be covered, since the female human genome reference hg19 (excluding chromosome Y) that was used consists to 6.6% of masked regions. These results are also consistent with previously reported genomes [15, 19]. For the exomes, on average over 96% of the targeted regions were covered. The genomes yielded a mean value of 3,063,616 variants, which is in agreement with an estimated human nucleotide diversity of 0.1% [20]. The expected transition/transversion (Ti/Tv) ratio for genome-wide single nucleotide variants (SNVs) is 2.1 and approximately 2.8 for exons [21] and can be used as an indicator for the quality of SNV detection. Our genome-wide SNVs accurately matched these values, while those for exome-wide SNVs ranged between 2.6 and 2.8, and were therefore between the recommended values for genomes and exomes. This is to be expected, as the enriched targets do not exclusively consist of exons, but to a large part also include additional regions such as the untranslated regions (UTRs) of numerous genes. As an additional quality control for our variant calls, we calculated the SNV concordance in pairwise comparisons for all twin pairs. Greater than 93% concordance was achieved for the genomes of TP1 and greater than 99% for the corresponding HiSeq exomes. The overlapping genome and exome SNVs were more than 93% concordant. The exomes of TP2 and TP3 showed more than 96% pairwise concordance. These results additionally confirmed the monozygosity of all analyzed twin pairs and suggest an adequate data quality. Detailed numbers for the concordance in all datasets are illustrated in Additional file 1: Figure S1. The ratio of nonsynonymous to synonymous changes among rare (frequency <0.5%) variants typically ranges between 1 and 2, and among common variants between 0.5 and 1.5 [22]. For a typical whole genome resequencing project the estimated ratio is between 0.8 and 1.0 [21]. Our observed nonsynonymous to synonymous ratios between 0.8 and 0.9 (Table 1) confirm these observations. The numbers of non-frameshift and frameshift InDels and nonsense SNVs detected in our study average below those of the 1000 genomes project (estimation of 130 to 178 non-frameshift and 192 to 280 frameshift InDels and 67 to 100 nonsense SNVs) while we detected a slightly higher number of splice-site variants (28 to 45 splice-site variants estimated). These deviations may be attributable to differences in data generation or more stringent variant calling. We estimated the number of potentially deleterious and disease-related variants in our dataset by computing the number of variants classified as disease-causing (DM) in the Human Gene Mutation Database (HGMD Professional, version 2013.3). Even a healthy genome contains between 50 and 100 DM variants [21] and we observed no excess in our dataset. We detected between 630 and 767 missense variants predicted to be “deleterious” by SIFT [23] or “probably damaging” by PolyPhen2 [24] in each of our genomes and exomes. Missense variants with a Grantham score above 100, representing amino acid changes defined as moderately radical (>100) and radical (>150 [25]) have a higher likelihood of clinical consequences. We found 17 to 18% of all missense variants to fall into this category. A high PhyloP value illustrates a strong evolutionary conservation of the affected genomic position and most pathogenic missense variants have been shown to have a PhyloP score above 2.5 [26]. With approximately 7,700 highly conserved variants the genomes only display approximately four times the number found in the exomes, despite magnitude differences in total size. This is attributable to the higher conservation of exonic regions. As even the genome of a healthy person contains at least 100 loss-of-function variants [27], it is difficult to establish a connection between the large number of potentially deleterious variants and a higher susceptibility to CD.

**Figure 2:**
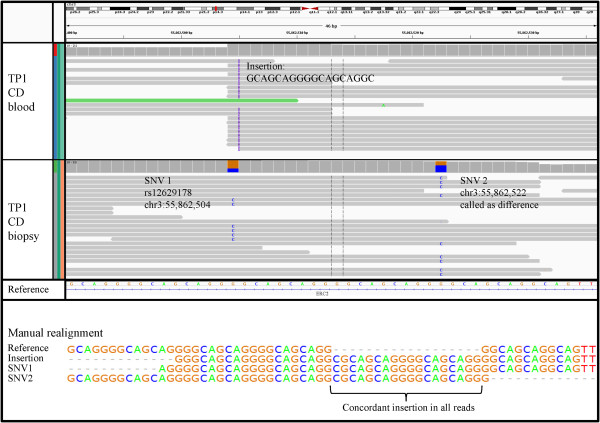
**Screenshot from IGV at a position called as a significant difference between the blood sample and biopsy exomes of the affected twin from TP1 (SNV2).** The alignment shows another SNV (SNV1) and an insertion nearby. Realignment of one read each shows identical alignability to the reference by introduction of a 19 bp insertion.

For the reliable detection of differences between co-twins we used three tools in parallel and separately evaluated the results to achieve the highest possible sensitivity and hence lowest false-negative ratio: SomaticSniper [28] employs a Bayesian comparison of the genotype likelihoods while VarScan [29] uses heuristic methods to test the significance of allele frequency differences using Fisher’s Exact Test. They both involve a direct comparison of the alignments of different samples to detect differences and were originally designed for tumor and normal pairs. The third tool pibase [30], previously released by our group, interrogates positions of interest in the alignment and performs a Fisher’s exact test incorporating different quality filters based on e.g. mapping quality, base quality and number of unique start points to determine the probability of differences. For TP1, with exome and genome data from blood samples as well as bowel biopsies available, we were able to scan for differences between the twins as well as tissue-specific mutations. For the exome data from the blood samples of TP2 and TP3, the comparison between affected and healthy twin was carried out accordingly. The comparison of the top 100 calls from the three methods showed only marginal overlap, confirming the results of previous studies [31]. A maximum of two variants were called by all three, the highest overlap was found between SomaticSniper and VarScan with four to 25 shared variants (Additional file 1: Figure S2 a-c). Somatic mutations with a p-value below 0.01 from Fisher’s exact test or in case of SomaticSniper a somatic score above 100, were manually checked through inspection of the alignments using the Integrative Genomics Viewer (IGV [32]). The majority of calls was already excluded at this stage. The false-positives were either attributable to false-negative calls in the healthy sample, mostly due to low quality alignments or low coverage, or they were caused by false-positive calls in the diseased sample, often due to alignment artifacts caused by nearby InDels. One example is shown in Figure 2 where a somatic mutation in the biopsy compared to the blood sample of the affected twin was called by SomaticSniper and VarScan on chromosome 3 at position 55,862,522 marked as “SNV2” in the intronic region of *ERC2*. The visual assessment of this region showed the occurrence of a second SNV and an insertion nearby in the alignments of the two samples. Manual realignment of reads containing one of the three variants each confirmed that the reads could all be aligned by introducing a 19 bp insertion (‘CGCAGCAGGGGCAGCAGGG’) compared to the reference sequence, confirming identical DNA sequences in both samples. For the pairwise comparisons in TP1, where four genomes and exomes were sequenced, the two samples not involved in the comparison were always available for further clarification, e.g. a variant detected in the blood sample of the CD twin and not in the blood sample of the healthy twin is highly unlikely to be a somatic mutation if we can also detect it in the bowel biopsy of the healthy twin. We were additionally able to cross-check exonic mutations from the exomes and genomes of the same samples. In total we visually inspected more than 2,200 potential somatic mutations for all three twin pairs (Additional file 1: Table S2). Following these steps, we excluded all candidates for TP1 and selected 15 variants from TP2 and TP3 for validation through Sanger sequencing. However, none of the variants could be confirmed as discordant as they were found to be either false-positive calls in one sample or present in both samples.

For TP1 we also performed differential copy number variation analysis. The genomes showed 128 CNVs potentially present only in the affected twin after filtering. These were visually inspected in the alignments and compared to the CNVs called for the exomes. The pairwise comparison of exome CNVs from blood samples did not show any region above the filtering cutoffs while the biopsies showed a deletion on chromosome 11 for the exome data. However, this region does not show up in the comparison of blood and biopsy of either twin, nor does it show up in the blood comparison or the genomic CNVs, and there are not sufficient SNVs to support a loss of heterozygosity (LOH) in the region. Therefore, none of the observed potential copy number losses or gains differing between twins or tissues showed sufficient support to be deemed of interest for follow-up analysis.

**Figure 3:**
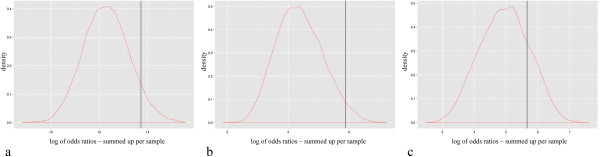
**Sum of odds ratios for the three twin pairs at 133 susceptibility loci for CD and IBD compared to 1920 healthy controls.** The vertical line represents the value of the respective twin pair. **a**: Calculated for all 133 SNVs in TP1. **b** and **c**: Calculated for 44 and 40 SNVs where genotypes were available for TP2 and TP3, respectively.

We estimated the genetic susceptibility of the twin pairs to CD by computing the genotypes of the genomes of TP1 and also, where available, those from the exomes of TP2 and TP3 at the identified CD risk loci ([1] Additional file 1: Table S3). The overall risk, represented by the summed up logarithmic odds ratios for the 133 CD risk alleles present in the twins, showed a higher mutation load in the three twin pairs in direct comparison to the distribution of the calculated overall risk of 1,920 healthy individuals genotyped on the Immunochip (Figure 3 a-c). This indicates that although genetic differences between the co-twins were not detected, a certain proportion of genetic susceptibility may still play a role for the development of CD in the affected twins and the discordance between twins is most likely attributable to environmental factors.

## Discussion

We here report the first whole genome and exome sequences of MZ twin pairs discordant for CD. We detected no reproducible differences within the twin pairs. Based on the good data quality and a high genotype concordance between samples and different methods, our data provided a solid foundation for the detection of somatic mutations between the co-twins. The low overlap between the three used tools for the detection of differences argues against the presence of somatic mutations. We therefore assume a high probability of the analyzed genomes and exomes being truly genetically identical. However, in spite of adequate data quality and the thorough analysis methods, the probability remains that we overlooked existing differences. The samples sequenced for TP1 consisted of blood samples as well as bowel biopsies for genomes and exomes. We decided on this approach since it allows for an exceptionally thorough look at the genetic data and offers the unique possibility of identifying tissue-specific somatic mosaicisms, especially in the tissue of interest for the particular disease. But the analysis of biopsy-derived DNA also involved a certain trade-off. The low yield of DNA extracted from a single biopsy required the pooling of DNA from several biopsies for the sequencing. A somatic mosaicism may not have been present in all biopsies used for sequencing and it is unlikely that we would have been able to detect mutations present in only a small fraction of the sequenced samples and if so, validation through Sanger sequencing would present another challenge. On the other hand, mutations present only in some regions of the bowel would be rather unlikely to have an effect on the development of Crohn’s disease. In the future, this problem may best be handled through the application of single cell sequencing [33], where the resulting genome truly represents the genome of a single cell and not the pool of what may possibly be several mosaics. This also applies to tumor genome resequencing where similar single-cell-based approaches are currently envisioned. For TP1, the biopsies were taken from affected regions in the colon, however the primarily ileocaecal disease localization in the affected twin may be an issue for finding somatic mutations. It is questionable if the affected twin from pair 1 represents an ideal candidate for a genetic study. With an age of onset of 45, only a few short hospital stays and no surgery needed so far, the course of the disease is comparatively mild and hence the genetic contribution may be low. However, the availability of the bowel biopsies from both twins considerably limited the number of possible twin pairs. In turn, we chose a lower age of onset and more severe disease course for TP2 and TP3, for whom only blood samples were available and subjected to sequencing. The three twin pairs chosen for this project therefore constitute the best possible trade-off between sample suitability and availability.

The theoretical probability that an offspring of a diamniotic MZ twin father carries a germline mutation that can be detected by allele-specific PCR in a sperm sample of their biological father, but not of his twin brother was defined at approximately 83% [34]. This thought experiment assumes circumstances clearly differing from the situation with our three female twin pairs, but nonetheless this estimated value may give an idea of the probability of finding differences in our twin pairs. Essentially, an experimental test of this theory in one male twin pair did detect five differences in genomes from sperm-derived DNA [35]. Only one of these SNVs however was also detected in the according blood samples, suggesting much variation in the detectable number of differences, also depending on the tissue type sequenced. In this study we focused solely on the detection of genetic differences between MZ twins for finding novel candidates for CD susceptibility. However, several other mechanisms may also be involved in disease discordance. Bowel biopsies of MZ twins discordant for ulcerative colitis, from the same cohort as in this study, were shown to differ in their DNA methylation patterns and exhibit differential gene expression [7], suggesting a role in disease development and yielding several novel candidate genes. For psoriasis, another disease involving chronic inflammation, DNA methylation and gene expression data in MZ discordant twins showed no differences between co-twins when analyzed separately. However, a combined analysis identified genes where differences in DNA methylation between unaffected and affected twins correlated with differences in gene expression [36] and hereby identified several known and novel susceptibility genes. In contrast, sequencing of the genomes of one twin pair and transcriptome sequencing, SNP chip and methylation chip data of three discordant twin pairs revealed no differences between co-twins discordant for multiple sclerosis [17].

## Conclusions

Our results are in accordance with previously published results and suggest that it is unlikely that somatic mutations have a substantial impact on the development of Crohn’s disease, yet we cannot completely exclude the possibility of having missed existing somatic mutations in the three twin pairs examined. Moreover, our study provides an analytical example framework to perform mosaicism detection and hints at potential weaknesses of existing variant detection tools. In the future, systematic transcriptome, methylome and microbiome analyses on MZ discordant twins using Next Generation Sequencing technologies are the way forward and can further elucidate the role of differential gene expression and differences in the microbial composition of the bowel in disease discordance.

## Methods

### Samples

The monozygotic twin pairs sequenced in this study were recruited as part of the cohort described in Spehlmann *et al.* [18]. The study setup was approved by the Bioethical Committee of the University of Kiel. All patients gave written informed consent before data and biomaterials were collected. Biopsies were taken endoscopically from a defined area of the colon, and immediately snap-frozen in liquid nitrogen. DNA was extracted from the biopsies using the QIAamp Tissue DNA preparation kit (Qiagen, Hilden, Germany). Genomic DNA was extracted from blood using the Invitek kit (Invitek, Berlin, Germany) and each DNA sample was evaluated by gel electrophoresis for the presence of high-molecular weight DNA.

### Sequencing

The four genomes of TP1 were sequenced on an Applied Biosystems SOLiD v.3+ on four to five whole slides per sample using a combination of paired-end (50 and 25 or 35 bp read length) and 1 kb insert mate-pair libraries (50 and 35 bp read length) (Additional file 1: Table S1). The four exomes of TP1 were enriched using Illumina’s TruSeq exome kit (Illumina, San Diego, CA, USA) according to the manufacturer’s instructions and sequenced together on one lane of an Illumina HiSeq2000 with 100 bp paired-end reads. The four exomes of TP2 and TP3 were enriched using the SureSelect Human All Exon kit v.2 (Agilent Technologies, Santa Clara, CA, USA) and sequenced with 50 and 35 bp paired-end reads on one quarter slide of the SOLiD v3+ each.

### Mapping and variant calling

The SOLiD reads were mapped using ABI’s Bioscope v1.2 software, for the Illumina HiSeq data we used BWA [37]. Further processing involved formatting with SAMtools [38] duplicate removal, local realignment around InDels, base quality score recalibration and coverage calculation using Picard (http://picard.sourceforge.net), BEDtools [39] and GATK [40], which was also used for variant detection. We applied our in-house tool *snp*Acts (http://snpacts.ikmb.uni-kiel.de/) for variant annotation, computation of concordance and their illustration in Venn diagrams.

### Detection of somatic mutations

For the genomes and exomes of TP1, three pairwise comparisons were carried out: CD biopsy vs. CD blood, CD blood vs. healthy blood and CD biopsy vs. healthy biopsy. We used SomaticSniper v0.7.4 [28] for all genomes and exomes, filtering reads with mapping quality below 10. The somatic score calculated by SomaticSniper represents the phred-scaled probability that the two genotypes are different and ranges between 0 and 255. We manually checked those with a somatic score above 100 (representing a probability of 1-10^(100/-10)^ for a difference between samples). For the comparison using pibase v.1.4.5 [30] we used all variant positions called for the respective exomes, while for the genomes we reduced the number of query positions by using the variants not called in all four and not called in both biopsy or blood samples and with a maximum 1000 genomes frequency of 20%. We then used *pibase_bamref* to query the positions in the bam files, *pibase_consensus* to compute the genotypes and *pibase_fisherdiff* for the pairwise comparison of samples using a Fisher’s exact test. All variants with p-values below 0.01 were manually inspected. The third tool for the detection of differences was VarScan v2.3.2. [29]. However, the results for the genome data indicated problems with the compatibility with SOLiD data mapped with BioScope, most likely due to the library types used, including different read lengths and insert sizes. We therefore excluded the VarScan results for the genomes. For the remaining comparisons we visually inspected somatic SNVs with p-values below 0.01 and no occurrence of the variant allele in the second sample. Additional file 1: Table S2 summarizes the number of inspected sites for all comparisons with the three tools and the concordances between the three methods are shown as Venn diagrams in Additional file 1: Figure S2 a-c.

CNVs in the genome data were detected using RDXplorer [41] and filtered with a customized pipeline. We narrowed down our selection the CNVs called in both samples of the affected twin but not in those of the healthy twin to find differences. We used BEDtools to detect CNVs reciprocally overlapping by at least 90% in the blood sample and biopsy of the affected (18,680 CNVs remaining), but not overlapping CNVs in the two samples from the healthy twin (1,235 remaining). We removed likely false-positive copy number loss calls locating to regions often experiencing low coverage due to mappability issues in non-unique genomic regions included in the “Mappability or Uniqueness of Reference Genome from ENCODE” track from UCSC [42] calculated for the uniqueness of the reference in 35 bp Windows (ftp://hgdownload.soe.ucsc.edu/goldenPath/hg19/encodeDCC/wgEncodeMapability/). For the remaining 128 CNVs potentially present only in the affected twin we added annotations from CCDS genes and known CNVs from the Database of Genomic Variants (DGV [43]) followed by manual inspection in the alignments in the Integrative Genomics Viewer (IGV [32]). For the CNVs in the exome data of TP1 we applied the in-house CNV calling pipeline, *clin*CNV (S. Ossowski, *personal communication*). This tool leverages the depth of coverage from multiple samples, and from paired samples (e.g. normal/tumor pairs) to identify copy number variable regions. Potential CNV regions were filtered based on number of 100 bp windows per region, percentage of windows with a positive loss or gain call, and average ratio against 58 healthy background control samples (20 baits, 80% support, somatic ratio <0.62 for losses or >1.38 for gains).

### Estimation of CD susceptibility

The susceptibility estimation was based on data from the Immunochip project of the International IBD Genetics Consortium (IIBDGC; http://www.ibdgenetics.org). The genotypes of 1,920 healthy German controls were extracted from the data set release 5 (MSControl_QC_Version3) and filtered down to the SNVs associated with CD by Jostins el al. [1] using the PLINK software [44]. The original 163 SNVs displaying association with CD, UC and IBD were based on release 4 of the data set. We excluded those exclusively associated with UC and another seven were removed in release 5 due to quality control filtering. For our dataset, the remaining 133 SNVs were used for TP1, of which 27 were exclusively associated with CD. For TP2 and TP3 the control dataset was reduced to those SNVs where genotypes were available from the exomes, resulting in a total of 44 and 40 SNVs, respectively (Additional file 1: Table S3). The risk alleles and odds ratios (OR) calculated by Jostins *et al.* were used for the allelic carriership based model: For each occurring risk allele in the twin pairs and the controls the logarithmic ORs were summed up. The distribution of the sum was plotted using R scripts [45], and the particular location for the analyzed twin pair was marked.

### Availability of supporting data

The genome and exome sequencing data supporting the results of this article are available upon request.

## Electronic supplementary material

Additional file 1: **Supplementary information: A: clinical history of the three monozygotic discordant twin pairs B: Supplementary Figures S1-S2 C: Supplementary Tables S1-S3.** (PDF 884 KB)
