# Supplementary material for: Whole genome and exome sequencing of monozygotic twins discordant for Crohn’s disease
Source: BMC Genomics. 2014 Jul 5;15(1):564. doi: 10.1186/1471-2164-15-564 (PMC4102722; doi:10.1186/1471-2164-15-564)
Supplement: Supplementary file 1 — Additional file 1: Supplementary information: A: clinical history of the three monozygotic discordant twin pairs B: Supplementary Figures S1-S2 C: Supplementary Tables S1-S3. (PDF 884 KB) [file 12864_2014_6260_MOESM1_ESM.pdf]

Supplementary Information for

Whole genome and exome sequencing of monozygotic twins discordant for Crohn's disease

Britt-Sabina Petersen[1§], Martina E. Spehlmann[2], Andreas Raedler[3], Björn Stade[1], Ingo Thomsen[1], Raquel Rabionet[4], Philip Rosenstiel[1], Stefan Schreiber[2], Andre Franke[1]

[1]Institute of Clinical Molecular Biology, Christian-Albrechts-University of Kiel, Germany

[2]Department of Internal Medicine, University Hospital Schleswig Holstein, Kiel, Germany

[3]Department of Internal Medicine II, Gastroenterology, Asklepios Westklinikum Hamburg, Hamburg, Germany

[4]Genomics and Disease Group, Center for Genomic Regulation, Barcelona, Spain

[§]Correspondence should be addressed to:

Britt-Sabina Petersen

Institute of Clinical Molecular Biology

Christian-Albrechts-University of Kiel

Schittenhelmstraße 12

24105 Kiel, Germany

phone: +49 431 597-1415

fax: +49 431 597-5788

email: b.petersen@ikmb.uni-kiel.de

A. Clinical history of three monozygotic discordant twin pairs

B. Supplementary Figures

C. Supplementary Tables

A. Clinical history of three monozygotic discordant twin pairs

In all cases, the twins were female and co-twins grew up together with high sanitary comfort during childhood. All affected twins presented with ileocaecal disease, confirmed by both endoscopy and histopathology.

The first twin pair (TP1) was 63 years old at time of first recruitment. At the time, the diseased twin had been suffering from Crohn´s disease for 17 years without fistula or stenosis, but with extraintestinal manifestations in form of arthritis. Surgery was not necessary and hospital stays were limited to a total of 1-3 months during time of disease. Total time of steroid intake was more than a year and flares occurred approximately once per year. Medications taken since disease onset include steroids, mesalazine, budesonide and infliximab the latter being the one she is currently treated with. Apart from an allergy against pollen she had no other known diseases additional to the intestinal disease. The healthy twin did not suffer from any diseases but had infectious urethritis in 2006.

The second twin pair (TP2) was 45 years old at time of recruitment. The affected twin had been suffering from Crohn´s disease for 25 years and presented with anal fistula to the skin. Arthritis was diagnosed as an extraintestinal manifestation in the patient. The patient had to undergo emergency gut surgery due to ileal perforation in the ninth year of disease. Hospitalization was necessary approximately once per year since disease onset and she spent more than 6 months overall in the hospital. Steroids were administered for up to one year during the course of the disease but flares have not occurred regularly anymore since surgery. Medications taken so far include steroids and mesalazine. At time of sample collection however, no medications were administered. She was a smoker at time of diagnosis but not at time of sample collection and her healthy co-twin also smoked. No diseases apart from

Crohn`s disease exist. The healthy co-twin did not suffer from any known diseases and showed no signs of intestinal disease.

The third twin pair (TP3) was 32 years old at the time of first participation in the study. The diseased twin had been diagnosed with Crohn´s disease 20 years before. The patient presented with fistula (both anal and inguinal) and had been admitted to the hospital less than every 2 years for a total of 1-3 months since the onset of disease. Frequency of flares was 1 to 3 per year. She was a smoker at the time of sample collection but not before disease onset. The affected twin did not suffer from any diseases apart from an allergy to pollen and the described IBD. At the time of sample collection, the patient was medicated with mesalazine only. She had taken steroids for a total of one year in the past. The healthy co-twin never smoked and did not suffer from any diseases apart from hypothyroidism caused by lack of iodine.

B. Supplementary Figures

Supplementary Figure S1 - Venn diagrams for the concordance of all called SNVs in genomes and exomes of the three twin pairs.

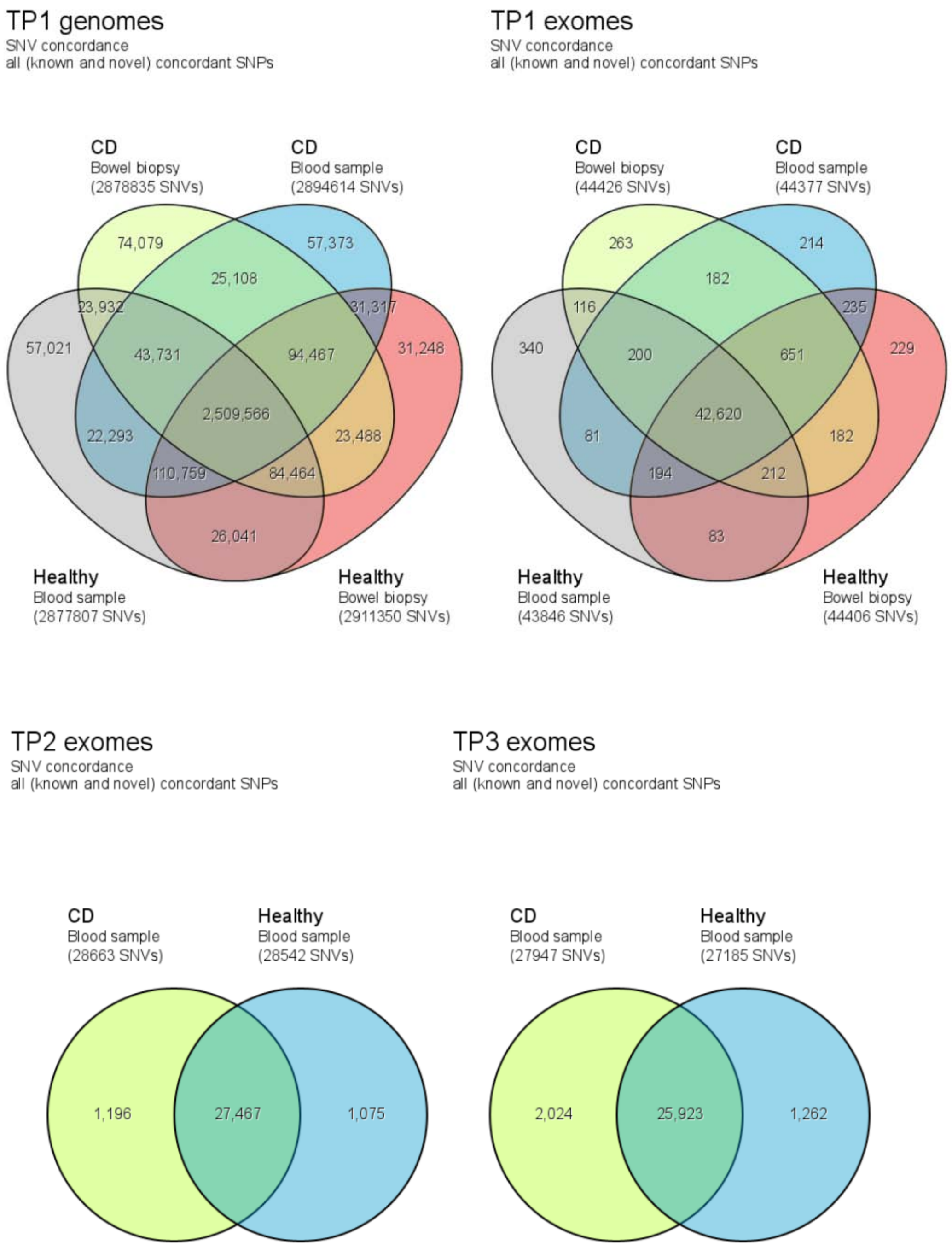

Supplementary Figure S2 - Venn diagrams illustrating the overlap between the top 100 of the somatic mutation callers used to detect differences. a: Overlap for the three comparisons performed for the four genomes of TP1 using pibase and SomaticSniper**. b:** Overlap for the three comparisons performed for the four exomes of TP1 using pibase, SomaticSniper and VarScan. **c:** Overlap for the comparisons performed for the blood samples of TP2 and TP3 using pibase, SomaticSniper and VarScan. Here, pibase called only 18 and 19 significant differences, respectively.

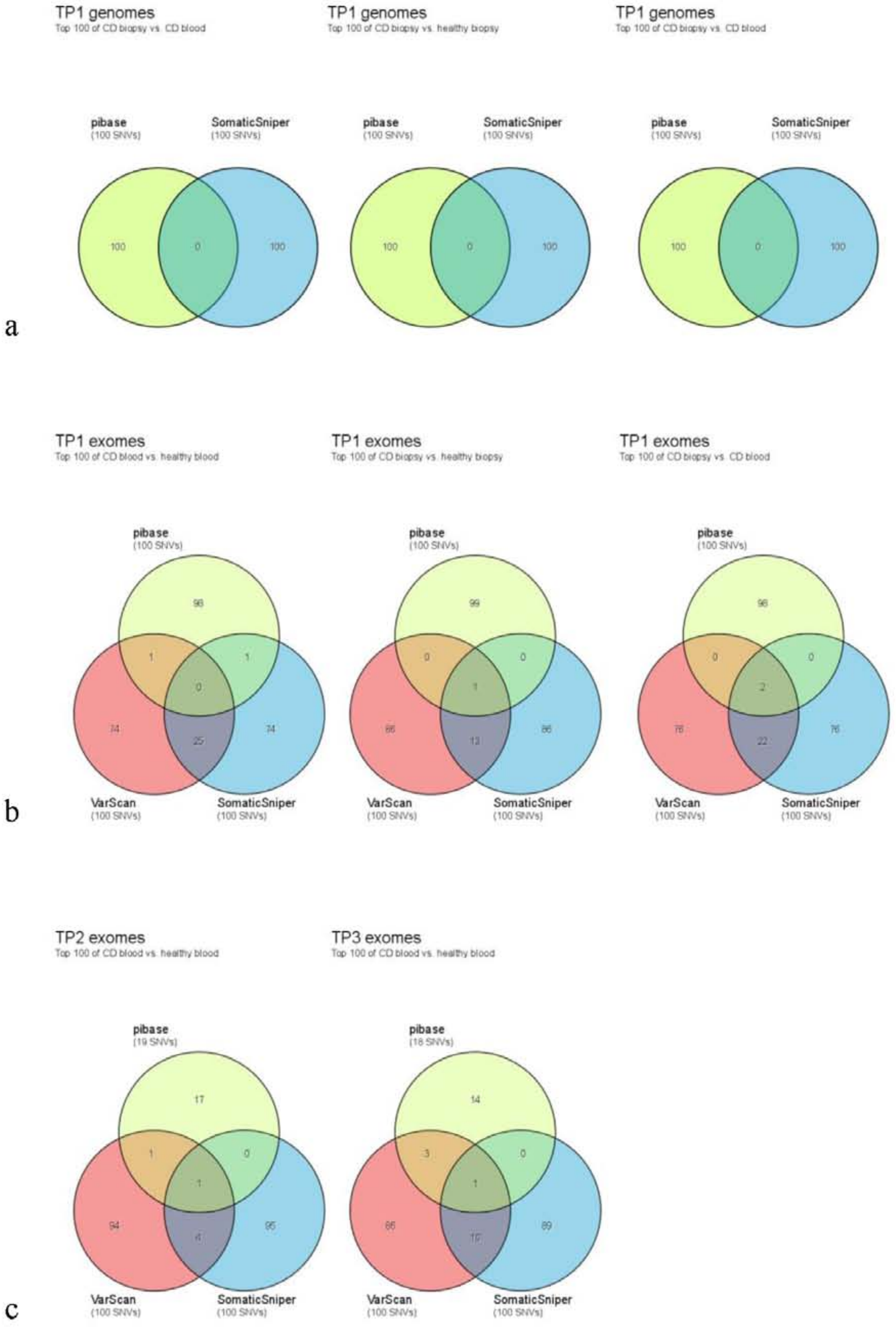

TP1 genomes
Top 100 of CD biopsy vs. CD blood
pibase
(100 SNVs)
SomaticSniper
(100 SNVs)
100
0
100
TP1 genomes
Top 100 of CD biopsy vs. healthy biopsy
pibase
(100 SNVs)
SomaticSniper
(100 SNVs)
100
0
100
TP1 genomes
Top 100 of CD biopsy vs. CD blood
pibase
(100 SNVs)
SomaticSniper
(100 SNVs)
100
0
100
a
TP1 exomes
Top 100 of CD blood vs. healthy blood
pibase
(100 SNVs)
98
1
1
0
74
25
74
VarScan
(100 SNVs)
SomaticSniper
(100 SNVs)
TP1 exomes
Top 100 of CD biopsy vs. healthy biopsy
pibase
(100 SNVs)
99
0
0
1
86
13
86
VarScan
(100 SNVs)
SomaticSniper
(100 SNVs)
TP1 exomes
Top 100 of CD biopsy vs. CD blood
pibase
(100 SNVs)
96
0
0
2
78
22
76
VarScan
(100 SNVs)
SomaticSniper
(100 SNVs)
b
TP2 exomes
Top 100 of CD blood vs. healthy blood
pibase
(19 SNVs)
17
1
0
1
94
4
95
VarScan
(100 SNVs)
SomaticSniper
(100 SNVs)
TP3 exomes
Top 100 of CD blood vs. healthy blood
pibase
(18 SNVs)
14
3
0
1
86
89
VarScan
(100 SNVs)
SomaticSniper
(100 SNVs)
c

C. Supplementary Tables

Supplementary Table S1: Libraries, sequencing and mapping details of the four genomes of TP1.

Sample type, individual	Library no.	Library type & sequencing length (bp)	First read in pair		Second read in pair		All reads	
			No. of reads	Mapped reads (%)	No. of reads	Mapped reads (%)	No. of reads	Mapped reads
Blood, CD	961	Paired-end 50-25	519,688,537	398,750,963 (76.73)	519,688,537	269,057,017 (51.77)	1,039,377,074	667,807,980
	961	Paired-end 50-35	559,495,443	411,013,028 (73.46)	559,496,019	285,756,172 (51.07)	1,118,991,462	696,769,200
	A0011	Mate-pair 50-50	589,392,975	278,754,706 (47.30)	589,414,032	451,927,364 (76.67)	1,178,807,007	730,682,070
	A0011	Mate-pair 50-50	617,629,481	392,309,644 (63.52)	617,629,481	438,083,184 (70.93)	1,235,258,962	830,392,828
	**Total**		2,286,206,436	1,480,828,341 (65.25)	2,286,228,069	[illegible],444,823,737 (62.61)	4,572,434,505	2,925,652,078
Biopsy, CD	773	Paired-end 50-25	484,234,252	381,636,788 (78.81)	452,073,774	141,160,435 (31.23)	936,308,026	522,797,223
	960	Paired-end 50-25	514,575,922	378,298,973 (73.52)	514,575,922	254,289,973 (49.42)	1,029,151,844	632,588,946
	960	Paired-end 50-35	538,252,530	270,516,130 (50.26)	538,252,530	202,613,709 (37.64)	1,076,505,060	473,129,839
	5143	Mate-pair 50-50	585,483,136	261,711,344 (44.70)	585,484,757	438,112,561 (74.83)	1,170,967,893	699,823,905
	5143	Mate-pair 50-50	553,016,065	234,307,685 (42.37)	553,016,311	367,414,882 (66.44)	1,106,032,376	601,722,567
	**Total**		2,675,561,905	1,526,470,920 (57.93)	2,643,403,294	[illegible],403,591,560 (51.91)	5,318,965,199	2,930,062,480
Blood, healthy	772	Paired-end 50-25	492,817,237	374,894,708 (76.07)	486,480,725	186,502,155 (38.34)	979,297,962	561,396,863
	963	Paired-end 50-25	457,943,882	305,183,001 (66.64)	457,943,882	199,190,271 (43.50)	915,887,764	504,373,272
	963	Paired-end 50-35	600,734,375	417,317,857 (69.47)	600,735,043	304,154,032 (50.63)	1,201,469,418	721,471,889
	A0012	Mate-pair 50-50	618,376,260	354,407,446 (57.31)	618,376,260	480,553,182 (77.71)	1,236,752,520	834,960,628
	A0012	Mate-pair 50-50	526,061,054	338,160,756 (64.28)	526,082,029	397,872,348 (75.63)	1,052,143,083	736,033,104
	**Total**		2,695,932,808	1,789,963,768 (66.75)	2,689,617,939	[illegible],568,271,988 (57.16)	5,385,550,747	3,358,235,756
Biopsy, healthy	962	Paired-end 50-25	549,371,666	384,274,533 (69.95)	549,371,811	265,723,189 (48.37)	1,098,743,477	649,997,722
	962	Paired-end 50-35	565,612,315	402,419,332 (71.15)	565,612,315	256,739,941 (45.39)	1,131,224,630	659,159,273
	962	Paired-end 50-35	629,910,387	410,094,578 (65.10)	629,910,387	107,353,553 (17.04)	1,259,820,774	517,448,131
	A1060	Paired-end 50-35	654,079,266	452,892,377 (69.24)	654,079,266	286,751,243 (43.84)	1,308,158,532	739,643,620
	**Total**		2,398,973,634	1,649,680,820 (68.86)	2,398,973,779	916,567,926 (38.66)	4,797,947,413	2,566,248,746

Supplementary Table S2: Number of significantly called differences by pairwise comparison and tool. Somatic score ≥ 100 for SomaticSniper, p-values ≤ 0.01 for pibase and VarScan.

Samples	Comparison	SomaticSniper	pibase	VarScan
**TP1 genomes**	CD biopsy - CD blood	24	285	-
	CD biopsy - healthy biopsy	66	626	-
	CD blood - healthy blood	75	319	-
**TP1 exomes**	CD biopsy - CD blood	30	119	91
	CD biopsy - healthy biopsy	22	129	164
	CD blood - healthy blood	9	120	63
**TP2 exomes**	CD blood - healthy blood	3	19	26
**TP3 exomes**	CD blood - healthy blood	8	18	70

Supplementary Table S3: Genotypes of the three twins pairs at CD and IBD susceptibility loci. Locus type: Susceptibility locus for CD or IBD; Chromosomal positions according to human genome build hg18; GT: genotype.

Locus type	Rs number	Immunochip ID	Chromosome	Position	Risk allele	Nonrisk allele	GT TP1	GT TP2	GT TP3
**CD**	rs17391694	rs17391694	1	78396214	C	T	CC	-	-
	rs6679677	imm_1_114105331	1	114105331	C	A	CC	-	-
	rs2641348	vh_1_120239407	1	120239407	A	G	AA	AA	AA
	rs7517810	imm_1_171120083	1	171120083	T	C	CC	CC	-
	rs1260326	rs1260326	2	27584444	T	C	TC	CC	TC
	rs10865331	imm_2_62404976	2	62404976	A	G	AA	-	-
	rs6716753	imm_2_230805373	2	230805373	C	T	TT	-	TT
	rs12994997	imm_2_233838242	2	233838242	A	G	AA	AG	GG
	rs7438704	rs7438704	4	48058002	G	A	GG	-	-
	rs13126505	imm_4_103084327	4	103084327	A	G	GG	-	-
	rs10065637	imm_5_55474608	5	55474608	C	T	CC	-	-
	rs10061469	1kg_5_72553904	5	72553904	T	C	CC	-	-
	rs12663356	rs12663356	6	21538707	C	T	CC	-	-
	rs2503322	rs2503322	6	127498953	G	A	GG	-	-
	rs13204742	imm_6_128287458	6	128287458	T	G	GG	GG	-
	rs212388	imm_6_159410424	6	159410424	C	T	CT	-	CC
	rs10486483	imm_7_26858965	7	26858965	A	G	GG	-	-
	rs864745	imm_7_28147081	7	28147081	T	C	CT	TT	-
	rs7015630	rs7015630	8	90945088	T	C	CT	-	-
	rs6651252	rs6651252	8	129636363	T	C	TT	-	-
	rs3764147	imm_13_43355925	13	43355925	G	A	AA	AG	AA
	rs16967103	imm_15_36686482	15	36686482	C	T	TT	-	-
	rs2945412	rs2945412	17	22867770	A	G	AG	-	AA
	rs2024092	1kg_19_1075031	19	1075031	A	G	GG	GG	GG
	rs4802307	rs4802307	19	51541646	G	T	GG	GG	GG
	rs516246	imm_19_53897984	19	53897984	T	C	CT	CT	TT
	rs2284553	rs2284553	21	33698565	G	A	AG	-	-
**IBD**	rs12103	rs12103	1	1237357	A	G	GG	GG	AA
	rs3766606	imm_1_7944784	1	7944784	G	T	GT	GG	-
	rs12568930	rs12568930	1	22574818	T	C	TT	-	-
	rs11209026	imm_1_67478546	1	67478546	G	A	GG	GG	GG
	rs2651244	rs2651244	1	70768150	G	A	AA	-	-
	rs4845604	rs4845604	1	150068304	G	A	GG	-	GG
	rs670523	imm_1_154145356	1	154145356	A	G	GG	-	-
	rs4656958	imm_1_159123588	1	159123588	G	A	AG	AA	GG
	rs2488389	imm_1_195897764	1	195897764	A	G	GG	-	-
	rs7554511	imm_1_199144185	1	199144185	C	A	CC	-	-
	rs3024505	imm_1_205006527	1	205006527	A	G	GG	GG	-
	rs13407913	rs13407913	2	24951148	G	A	AA	-	-
	rs925255	rs925255	2	28468298	C	T	CT	-	-
	rs10495903	1kg_2_43660422	2	43660422	T	C	CC	-	-
	rs7608910	imm_2_61058360	2	61058360	G	A	AG	-	AA
	rs6740462	imm_2_65520776	2	65520776	A	C	AA	-	-
	rs6708413	imm_2_102429801	2	102429801	G	A	AG	AG	AG
	rs2111485	imm_2_162818782	2	162818782	A	G	AG	-	-
	rs1517352	imm_2_191639709	2	191639709	C	A	AC	-	-
	rs2382817	seq-rs2382817	2	218859462	A	C	AC	-	-
	rs3749171	1kg_2_241218365	2	241218365	T	C	CC	CT	CT
	rs4256159	1kg_3_18742408	3	18742408	T	C	CC	TT	-
	rs3197999	imm_3_49696536	3	49696536	A	G	GG	AA	AG
	rs2457996	rs2457996	4	75075399	T	C	CT	CC	-
	rs7657746	imm_4_123381069	4	123381069	A	G	AA	-	AA
	rs2930047	rs2930047	5	10748526	C	T	CT	-	-
	rs11742570	imm_5_40446341	5	40446341	C	T	CT	TT	-
	rs1363907	imm_5_96278559	5	96278559	A	G	GG	-	-
	rs10051722	rs10051722	5	130131975	A	C	AC	-	-
	rs6863411	imm_5_141493388	5	141493388	T	A	AT	-	-
	rs11741861	imm_5_150258102	5	150258102	G	A	AA	AA	AG
	rs6871626	imm_5_158759370	5	158759370	A	C	AA	-	-
	rs4976646	rs4976646	5	176721176	A	G	AA	-	-
	rs17119	rs17119	6	14827475	A	G	AA	-	-
	rs9358372	imm_6_20920567	6	20920567	G	A	GG	-	GG
	rs1847472	imm_6_91029880	6	91029880	C	A	AC	-	CC
	rs7746082	imm_6_106541962	6	106541962	C	G	GG	-	-
	rs3851228	rs3851228	6	111954884	T	A	AA	-	-
	rs6920220	imm_6_138048197	6	138048197	A	G	AG	-	-

rs12199775	rs12199775	6	143940587	A	G	AA	-	-
rs1819333	imm_6_167293537	6	167293537	T	G	TT	-	-
rs1456896	imm_7_50275007	7	50275007	T	C	CT	CT	TT
rs9297145	rs9297145	7	98597053	C	A	AA	-	-
rs38911	rs38911	7	116682399	G	A	AA	GG	-
rs921720	ccc-8-126603853-A-G	8	126603853	G	A	GG	-	-
rs13277237	rs13277237	8	130673745	G	A	AA	-	-
rs10758669	imm_9_4971602	9	4971602	C	A	CC	-	-
rs4743820	rs4743820	9	92968237	T	C	CC	-	-
rs4246905	imm_9_116593070	9	116593070	C	T	CC	CT	CT
rs10781499	imm_9_138386226	9	138386226	A	G	AG	GG	AG
rs12722515	imm_10_6121236	10	6121236	C	A	CC	-	CC
rs1042058	1kg_10_30768107	10	30768107	C	T	CT	CC	TT
rs11010067	imm_10_35335437	10	35335437	G	C	CG	-	-
rs2790216	1kg_10_59667932	10	59667932	G	A	AG	-	-
rs10761659	imm_10_64115570	10	64115570	G	A	AA	AA	-
rs2227551	rs2227551	10	75339196	T	G	GT	GG	-
rs1250546	imm_10_80702538	10	80702538	A	G	AA	AA	-
rs7097656	rs7097656	10	82240811	C	T	CT	-	-
rs12778642	rs12778642	10	94454287	T	G	GT	-	-
rs4409764	imm_10_101274227	10	101274227	T	G	TT	-	-
rs907611	rs907611	11	1830648	A	G	AG	-	-
rs11229555	rs11229555	11	58165263	G	T	GT	-	-
rs11230563	imm_11_60532785	11	60532785	C	T	CC	CC	TT
rs174537	rs174537	11	61309256	T	G	TT	-	-
rs559928	rs559928	11	63906946	C	T	CC	CC	TT
rs568617	rs568617	11	65409818	T	C	CC	CC	CC
rs2155219	imm_11_75976842	11	75976842	T	G	GT	-	-
rs2226628	rs2226628	11	86769493	A	G	AA	-	-
rs566416	imm_11_118264820	11	118264820	T	G	TT	-	-
rs11054935	rs11054935	12	12540110	G	A	AG	-	-
rs11564258	imm_12_39078567	12	39078567	A	G	AG	-	-
rs11168249	rs11168249	12	46494635	C	T	TT	-	-
rs7134599	imm_12_66786342	12	66786342	A	G	GG	-	-
rs17085007	rs17085007	13	26429267	C	T	CT	TT	-
rs941823	rs941823	13	39911977	C	T	CT	-	TT
rs3742130	rs3742130	13	98705342	G	A	GG	GG	GG
rs194749	imm_14_68343658	14	68343658	C	T	CT	-	-
rs1569328	rs1569328	14	74811504	C	T	CC	-	-
rs8005161	1kg_14_87542348	14	87542348	T	C	CC	-	-
rs17293632	imm_15_65229650	15	65229650	T	C	TT	TT	CC
rs529866	rs529866	16	11280821	C	T	CC	-	-
rs7404095	rs7404095	16	23772091	C	T	CT	TT	-
rs26528	imm_16_28425210	16	28425210	C	T	CT	-	CC
rs2361755	imm_16_84567187	16	84567187	G	C	GG	-	-
rs3091315	1kg_17_29617778	17	29617778	A	G	AA	-	-
rs12946510	imm_17_35165903	17	35165903	T	C	CT	CC	-
rs12942547	imm_17_37781070	17	37781070	A	G	AG	AA	AA
rs1292053	rs1292053	17	55318319	G	A	AG	AG	AG
rs1893217	imm_18_12799340	18	12799340	G	A	AA	-	-
rs7240004	rs7240004	18	44649020	A	G	AG	AA	-
rs727088	imm_18_65681419	18	65681419	G	A	AG	-	-
rs11879191	imm_19_10373911	19	10373911	G	A	AG	-	GG
rs17694108	rs17694108	19	38423391	A	G	AG	-	-
rs1654644	seq-rs1654644	19	60065174	G	T	GT	-	TT
rs4243971	rs4243971	20	30313178	G	T	GT	-	-
rs6087990	rs6087990	20	30813569	C	T	TT	CC	-
rs6074022	imm_20_44173603	20	44173603	C	T	TT	-	TT
rs913678	rs913678	20	48388831	T	C	CT	-	
rs259964	rs259964	20	57257704	A	G	AA	AA	GG
rs6062504	rs6062504	20	61819351	G	A	GG	-	-
rs2823286	imm_21_15739809	21	15739809	G	A	GG	-	-
rs2836878	imm_21_39387404	21	39387404	G	A	GG	-	-
rs7282490	imm_21_44440169	21	44440169	G	A	AG	-	-
rs2266959	imm_22_20252904	22	20252904	T	G	GT	-	-
rs5763767	imm_22_28823882	22	28823882	A	G	GG	AA	-
rs2413583	imm_22_37989719	22	37989719	C	T	CT	-	-
